# Characterization of glycosylation regulator-mediated glycosylation modification patterns and tumor microenvironment infiltration in hepatocellular carcinoma

**DOI:** 10.3389/fgene.2022.1001901

**Published:** 2022-11-11

**Authors:** Linlin Zhao, Yang Guo, Zhanfeng Liu, Jing Ma, Yanfeng Peng, Dejun Zhang

**Affiliations:** ^1^ Research Center for High Altitude Medicine, Medical College of Qinghai University, Xining, China; ^2^ Department of General Surgery, The First People’s Hospital Xining City, Xining, China; ^3^ Key Laboratory of Application and Foundation for High-Altitude Medicine Research in Qinghai Province, Xining, China; ^4^ Qinghai-Utah Joint Research Key Laboratory for High Altitude Medicine, Xining, China; ^5^ College of Eco-Environmental Engineering, Qinghai University, Xining, China

**Keywords:** hepatocellular carcinoma, glycosylation, prognosis, immunocyte infiltration, sensitive drugs

## Abstract

**Background:** Previous studies have shown that glycosylation of proteins ofen plays an important role in HCC. However, the potential mechanism of glycosylation in HCC has not been described systematically.

**Methods:** We comprehensively evaluated the glycosylation patterns in HCC samples based on 43 glycosylation regulators, and annotated the modification patterns with the enrichment of immune cells and stromal cells. Considering the heterogeneity of HCC patients, the glycosylation score was constructed using single-sample gene set enrichment analysis (ssGSEA). We also explored the drugs that different HCC patients were sensitive to based on glycosylation mode and score.

**Results:** We identified three glycosylation-regulated gene subtypes. By annotating the subtypes, it was found that the glycosylation regulated gene subtypes was highly matched with three immunophenotypes of HCC (immune-inflamed, immune-excluded, and immune-desert), regardless of the characteristics of immune cell infiltration or prognosis. Based on the characteristic genes of glycosylation-regulated gene subtypes, we constructed a glycosylation-related model, and found that glycosylation-related model was highly consistent with the glycosylation regulated gene subtypes. The glycosylation score that evaluates the glycosylation characteristics of a single HCC sample has high prognostic value, and the prognosis of patients with high glycosylation score is significantly worse. Interestingly, we found that the glycosylation score was closely related to tumor node metastasis (TNM) staging. By applying glycosylation-regulated gene subtypes and glycosylation score to explore the sensitivity of different patients to anticancer drugs, it was found that the sensitivity of Thapsigargin, Shikonin, Embelin and Epothilone. B was closely related to the glycosylation mode.

**Conclusion:** This study reveals that the diversity of glycosylation patterns plays an important role in HCC. Therefore, evaluating the glycosylation patterns of patients with HCC will be helpful in identifying the characteristics of immune cell infiltration and selecting accurate treatment methods.

## 1 Introduction

Post-translational modification of proteins is the main factor that affects the functional diversity of the proteome. At present, post-translational modification of protein mainly includes phosphorylation, glycosylation, ubiquitination, nitrosylation, methylation, acetylation, lipidization, and proteolysis (Vu et al., 2018). Glycosylation, an important post-translational modification of proteins, plays an important role in various diseases, such as tumors, viral infections, and Alzheimer’s disease, mainly by affecting the stability of protein structures (Park, 2019; Tao and Huang, 2019; Liu et al., 2021). Abnormal glycosylation can affect the progression of a variety of tumors and usually involves the regulation of epithelial mesenchymal transformation and the immune microenvironment (Wattanavises et al., 2019; Zhang et al., 2020a). Therefore, an in-depth exploration of the role of these regulatory factors in hepatocellular carcinoma (HCC) will help reveal their mechanism of action.

Glycosylation of different proteins play different roles in HCC. For example, Hitomi et al. showed that N-glycosylation of fuc increases with the progression of HCC and can be used as an independent prognostic factor for HCC (Asazawa et al., 2015). A recent study on HCC showed that N-glycosylation of Ces1 can inhibit the proliferation of HCC cells (Na et al., 2020). Glycosylation of LAMP2 and SV40 serves as a protective mechanism against HCC (Pousset et al., 1997; Chiu et al., 2022). Glycosylation of HCC mostly occurs in oncogenes such as *AFP, AACT,* and *MDR*, which can be used as risk factors for HCC (Ledoux et al., 2003; Lee et al., 2022). Glycosylation of these genes is not only involved in the molecular mechanism of HCC progression but is also closely related to tumor drug resistance. In HCC, mRNA, miRNAs, and lncRNAs can participate in the regulation of glycosylation (Liu et al., 2017; Liu et al., 2018). Glycosylation of PD-L1 may lead to immunosuppression or inactivation (Huang et al., 2019). However, the role of glycosylation in the tumor microenvironment of HCC remains unclear.

Owing to experimental limitations, research in the field has been limited to one or two glycosylation regulators, even though the mechanism of glycosylation regulation in HCC is regulated by the interaction of many other glycosylation regulators in a highly coordinated manner. Therefore, a systematic understanding of the role of multiple glycosylation regulators is essential for studying glycosylation regulatory models in HCC. In this study, we integrated the genomic information of HCC samples in ‘The Cancer Genome Atlas’ (TCGA) and Gene Expression Omnibus (GEO) databases to comprehensively evaluate the glycosylation regulatory model and defined different subtypes using the enrichment of immune cells and stromal cells. We identified three glycosylation subtypes in this study and surprisingly found that the characteristics of these three subtypes were highly consistent with immune rejection, immune inflammation, and immune desert phenotypes. In addition, we constructed a glycosylation score for HCC to predict the prognosis and drug sensitivity of different patients.

## 2 Materials and methods

### 2.1 Hepatocellular carcinoma data set and preprocessing

**FIGURE 1:**
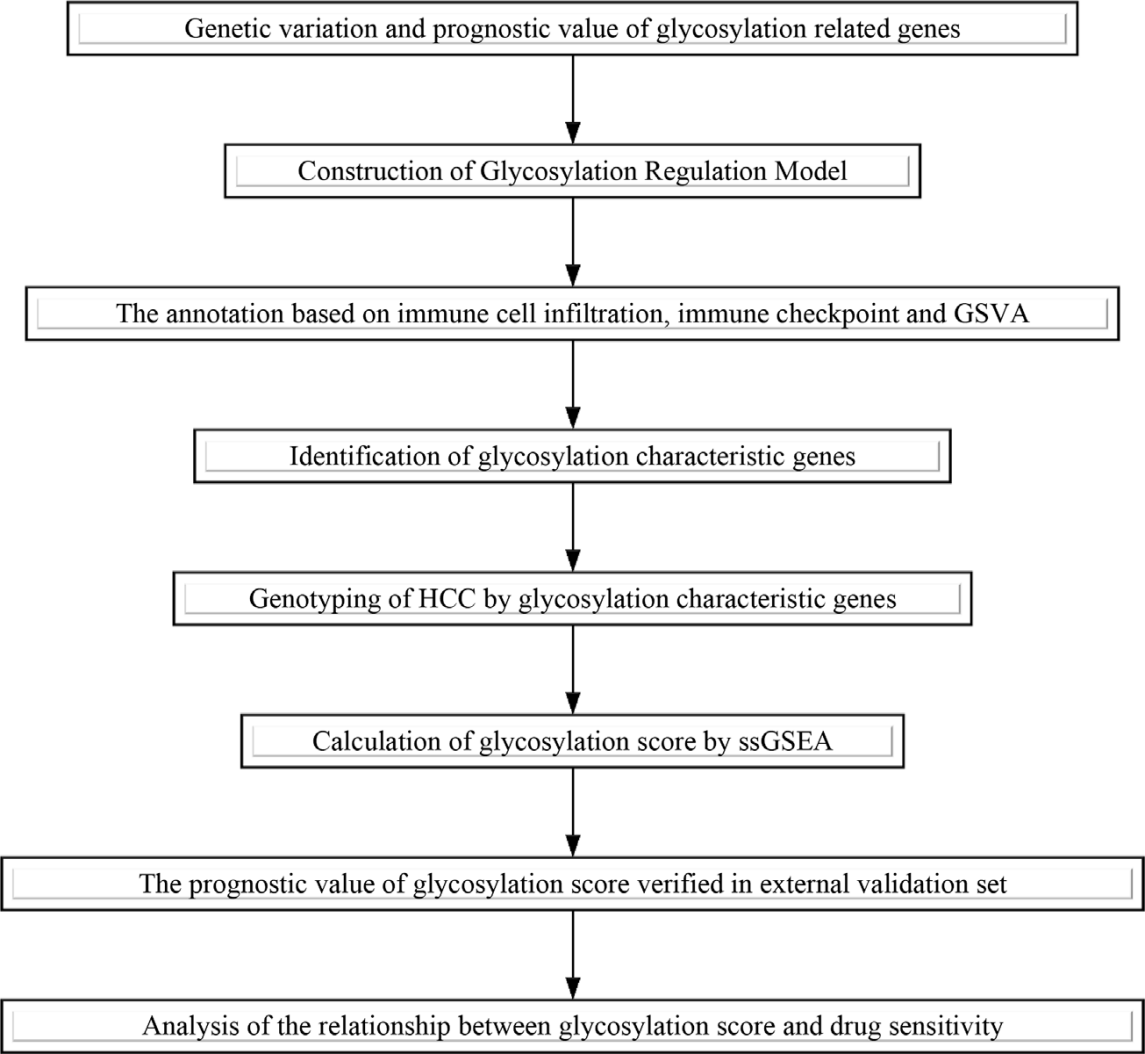
Flow-process diagram.

HCC datasets with gene expression data and complete clinical information were retrieved from TCGA and GEO databases. Finally, TCGA-LIHC, GSE76427, and GSE14322 were included in subsequent analysis. By deleting samples with missing clinical information in the dataset, TCGA-LIHC included 281 HCC samples. GSE76427 included microarray data of 115 HCC cases with complete clinical data. GSE14322 included 167 samples, including 115 cases of HCC and 52 cases of normal HCC. Gene annotation and data standardization were performed to obtain a standard gene expression matrix. Data on somatic mutations and copy number variation (CNV) of HCC were obtained from TCGA. The list of glycosylation regulators were summarized from previous literature and the molecular signature database V7.4 (Liberzon et al., 2015). The specific analysis process of this study is shown in Figure 1.

### 2.2 Unsupervised hierarchical clustering analysis of glycosylation regulators

Univariate Cox regression analysis was applied to screen for prognosis-related glycosylation regulatory genes, with a screening criteria of *p* < 0.05. The limma package was applied based on the GSE14322 cohort analysis of glycosylation regulatory genes differentially expressed in HCC and adjacent tissues. The intersection of the two was used to identify differentially expressed glycosylation regulatory genes with prognostic value for subsequent unsupervised cluster analysis. Before unsupervised clustering, the conditional survival curve was constructed using the surviviner and survival packages to check the availability of the data. Consensus clustering was applied to build a glycosylation regulatory gene model, and its clustering number and stability were determined by a consensus clustering algorithm (Timmerman et al., 2013). This process was performed on the ConsensusClusterPlus package of R version 4.1.2, which was repeated 1,000 times to ensure the stability of the model (Wilkerson and Hayes, 2010).

### 2.3 Verification of glycosylation regulated gene subtypes

To verify the prognostic value of different glycosylation regulatory genes, survival analysis of patients with different subtypes was performed using the surviviner and survival packages to compare the survival of different subtypes. Nomograms were constructed for clinical application based on age, sex, tumor node metastasis (TNM) stage, race, and glycosylation-regulated gene subtypes. A calibration curve was constructed to test the accuracy of the nomogram. This process was performed on the nomogramEx package.

### 2.4 Principal component analysis and calculation of glycosylation score

To further verify the subtypes of glycosylation regulatory genes, common differentially expressed genes among the different subtypes were analyzed using the limma package as glycosylation-related genes. Univariate Cox regression was used to screen for glycosylation-related genes with prognostic value. The advantage of PCA is that it can cluster genes with the largest contribution, whereas genes with smaller contributions are not included in the clustering process. In this study, PCA was used to conduct a secondary clustering of patients with HCC. Surviviner and survival packages were used to explore the survival of patients with different subtypes. Then, single-sample gene set enrichment analysis (ssGSEA) was used to calculate the glycosylation score for each sample (Yi et al., 2020). The relationship between the glycosylation score and different subtypes was explored to verify the predictive power of the glycosylation regulation model in HCC.

### 2.5 Analysis of immune cell infiltration and stromal cell enrichment

An increasing number of studies have shown that the tumor microenvironment plays an important role in the pathogenesis and drug resistance of tumors (Wang et al., 2021). Exploring the infiltration of immune cells in different glycosylation regulatory genes is not only helpful in the choice of immunotherapy for different patients, but also in defining different glycosylation subtypes. xCell is a new gene signature method based on the 1822 pure human cell-type transcriptome, which infers 64 immune and stromal cell types (Aran et al., 2017). To show the differences in immune cell infiltration and stromal cells in different subtypes, we used xCell to comprehensively analyze the distribution of immune and stromal cells in each subtype. This process was primarily performed on the IOBR package and was visualized using the pheatmap package.

### 2.6 Immune checkpoint analysis

The distribution of immune checkpoints is closely related to HCC (Xu et al., 2018). In this study, we selected common immune checkpoints for HCC, including CYBB, IDO1, KIR3DL1, HAVCR2, CD274, PDCD1, TIGIT, CTLA4, LAG3, BTLA, CD27, CD28, CD40, IL2RB, TNFRSF9, TNFRSF4, TNFRSF18, and ICOS. By verifying the expression of immune checkpoints in different subtypes, we can determine the responsiveness of patients with different subtypes to immunotherapy.

### 2.7 Gene set variation analysis

GSVA can calculate the pathway enrichment score of each sample according to the expression matrix and enrichment of the reaction pathway in the sample (Tao et al., 2021). The clusterProfiler package was used to calculate the path enrichment score of each sample and to construct the expression matrix of the pathway. The limma package was used for differential expression analysis to determine pathways with differences between different subtypes. In this study, to show the enrichment of pathways in different genotypes, we performed GSVA analysis on the subtypes based on the glycosylation-regulated gene model. h.all.v7.4. symbols.gmt was downloaded from the MSigDB database as the reference gene set, and *p* < 0.05 was considered statistically different.

### 2.8 Drug sensitivity analysis

The Genomics of Drug Sensitivity in Cancer (GDSC) database (Yang et al., 2013), which is designed to apply therapeutic biomarkers to improve the therapeutic efficacy in cancer patients, can detect drug sensitivity based on the genetic characteristics of different patients. In this study, to guide clinical medication based on the glycosylation regulatory gene subtype, we applied the pRRophetic package to identify sensitive drugs in patients with HCC with different glycosylation subtypes.

### 2.9 Statistical analyses

All statistical analyses were performed using R version 4.1.2. Measurement data between the two groups were tested for normality and homogeneity of variance and significant differences estimated using two-sample *t*-test if the two conditions were met, and a Wilcoxon rank-sum test otherwise. The measurement data among three groups were analyzed using ANOVA, and the Bonferroni test was used for comparison between the two groups. Statistical significance was set at *p* < 0.05.

## 3 Results

### 3.1 Genetic variation of glycosylation related genes in hepatocellular carcinoma

**FIGURE 2:**
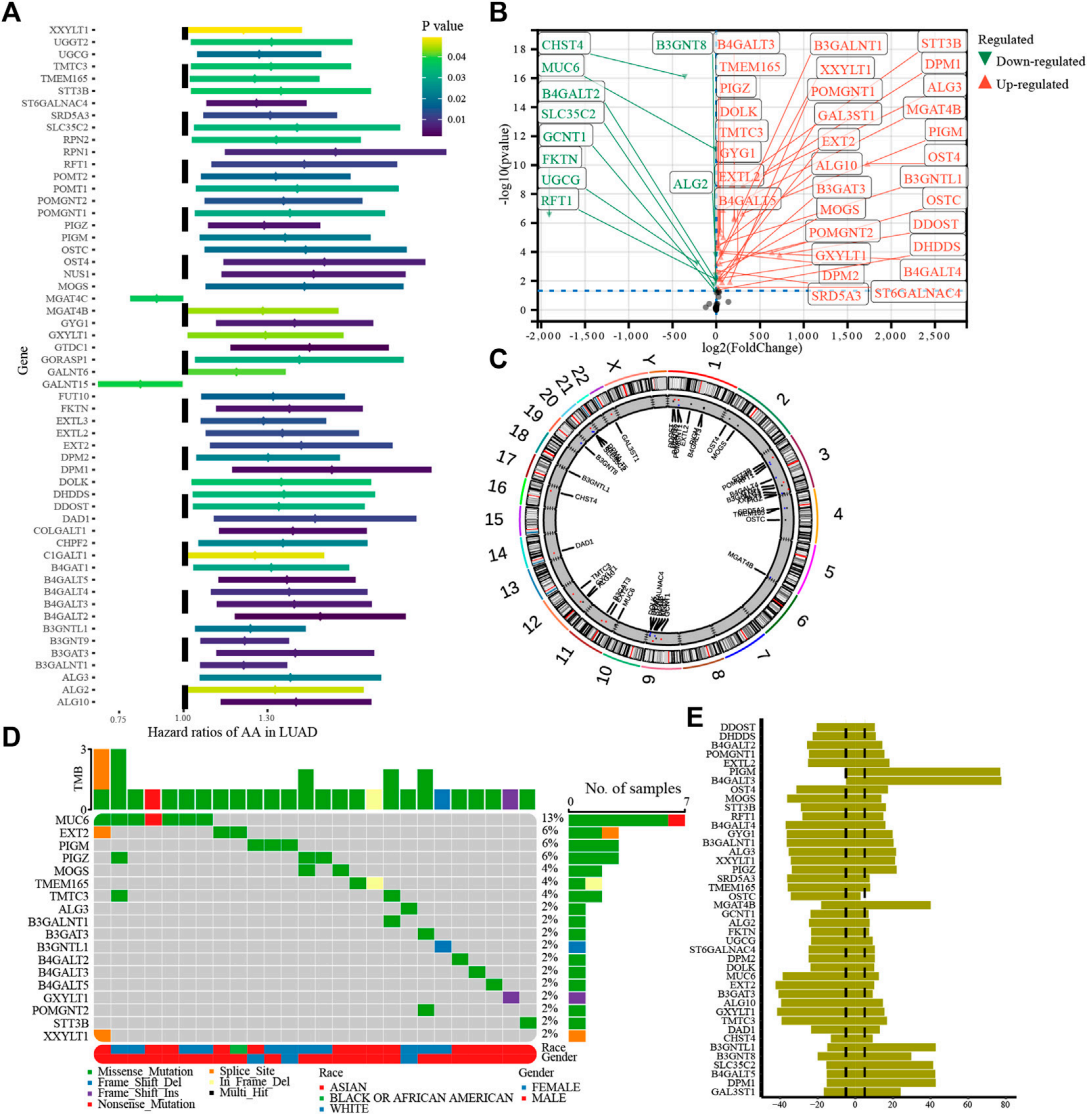
Genetic variation in glycosylation-regulatory genes in HCC. **(A)**. Univariate Cox regression analysis of glycosylation regulatory genes; **(B)**. Differential expression of glycosylation regulated genes with prognostic value; **(C)**. Location of 43 differentially expressed genes with prognostic value on the chromosome; **(D)**. Gene mutation of glycosylation regulator; **(E)**. CNV of glycosylation regulators.

This study analyzed a glycosylation gene modification model based on 255 glycosylation-regulatory genes (Supplementary Table S1). A total of 56 prognosis-related glycosylation regulatory genes (Figure 2A) were identified using univariate Cox regression analysis. Excepting that the high expression of *MGAT4C* and *GALNT15* suggested a better patient prognosis, all the other genes were risk factors for HCC. By differential expression analysis of the 56 prognosis-related genes, 43 genes were found to have significant differences between HCC and normal tissues (Figure 2B). The positions of 43 differentially expressed glycosylation regulators with prognostic value on chromosomes are shown in Figure 2C. Based on the analysis of 43 prognostic genes of mutation data in the TCGA database, 18 genes, namely *XXYLT1*, *STT3B*, *POMGNT2*, *GXYLT1*, *B4GALT5, B4GALT3*, *B4GALT2*, *B3GNTL1*, *B3GAT3*, *B3GALNT1*, *ALG3*, *TMTC3*, *TMEM165*, *MOGS*, *PIGZ*, *PIGM*, *EXT2*, and *MUC6* were mutated in HCC. Among them, *MUC6* had the highest mutation frequency (Figure 2D). Most of the mutations were missense, and MUC6 expression was significantly lower in HCC, suggesting that mutations in *MUC6* may affect MUC6 expression. CNV analysis suggested that amplified copy number variation appeared in *PIGM*, *B4GALT3*, and *MGAT4B*, and deletion CNV appeared in *MUC6*, *B3GAT3*, and *B4GALT4* (Figure 2E). Gene mutation and CNV analyses indicated that the CNV of the 43 glycosylation regulators was the main factor affecting their expression in HCC.

### 3.2 Modification models mediated by 43 glycosylation regulating genes

**FIGURE 3:**
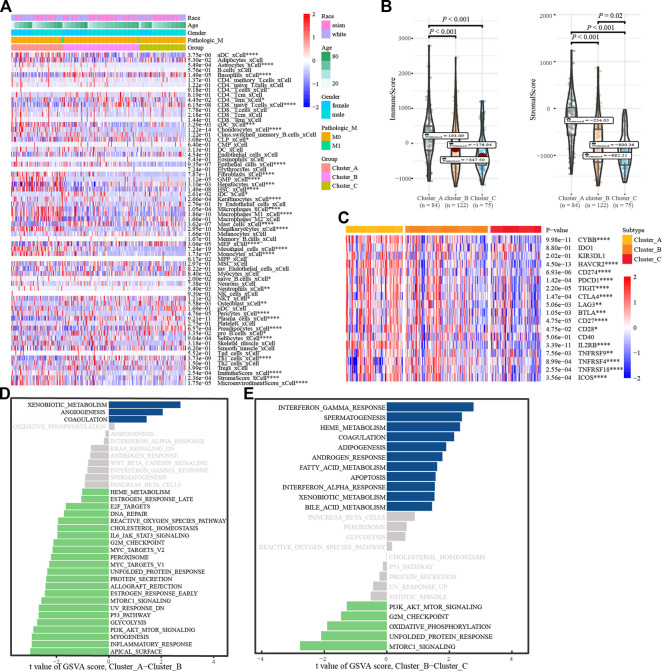
Glycosylation regulatory gene model. **(A)**. Conditional probability survival curve; **(B)**. Apply consensus clustering to construct a glycosylation regulator gene model; **(C)**. Kaplan-Meier survival analysis of the glycosylation regulator gene model; **(D)**. Calibration curve at 5 and 8 years; **(E)**. Nomogram.

The conditional probability survival results are shown in Figure 3A. The longer the survival time of patients with HCC, the higher the subsequent survival rate. The 5-year survival rate of patients increased by 66%, 75%, 81%, and 86%, respectively, from the overall survival rate of 49%. The conditional probability of survival was consistent with that of patients with HCC, indicating that the data could be used for subsequent analysis. Based on consensus clustering, HCC patients were divided into three subtypes (Figure 3B): female patients, T3-T4 patients, and M1 patients were most likely to be in Cluster_A, and M0 patients were not found in Cluster_C. Patients in Cluster_B had the best prognosis among the three glycosylation types, whereas the survival of patients in Cluster_A and Cluster_C was not significantly different (Figure 3C), which showed that the typing model was highly correlated with the TNM stage. Therefore, we constructed a nomogram based on the glycosylation regulatory gene model. The calibration curve showed the high accuracy of the nomogram (Figure 3D). Using the nomogram plot, we can predict patient survival at five and 8 years based on age, sex, TNM stage, ethnicity, and glycosylation regulatory gene model.

### 3.3 Annotations of glycosylation regulatory gene model

**FIGURE 4:**
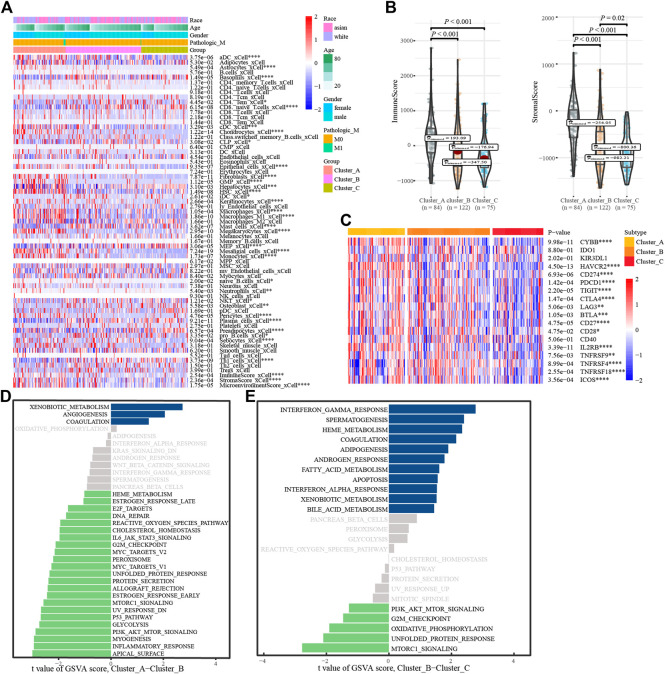
Annotation of the glycosylation regulatory gene models. **(A)**. Enrichment of immune cells and stromal cells in different subtypes; **(B)**. Differences in immune scores and stromal scores in different subtypes. **(C)**. Differential expression analysis of immune checkpoints in different subtypes; **(D)**. The GSVA of Cluster_A-Cluster_B; **(E)**. The GSVA of Cluster_B-Cluster_C.

To understand the biological behavior associated with the different glycosylation regulatory models, we annotated them using immune cell infiltration analysis, stromal cell enrichment analysis, immune score, stromal score, immune checkpoint, and GSVA. Immune cell infiltration analysis showed (Figure 4A) basophils_ xCell, and CD8. The naive T. Cells and macrophages_ M1_ Xcel and other immune cells in cluster_ A had the highest infiltration, whereas cluster_C had the lowest infiltration. The distribution of stromal cells, such as preadipocytes and hepatocytes, in different subtypes was consistent with that of the immune cells. Surprisingly, stromal and immune scores also tended to be consistent across the subtypes (Figure 4B). Analysis of the expression of immune checkpoints in different glycosylation regulatory gene subtypes (Figure 4C) revealed that all the immune checkpoints, except IDO1, KIR3DL1, and CD40, had the highest expression in Cluster_A and the lowest expression in Cluster_C. Based on the above analysis (Figures 4A–C), we classified Cluster_A as an immune rejection phenotype, which was characterized by the highest immune cell infiltration and stroma activation. Cluster_B was classified as an immune-inflammatory phenotype, characterized by partial immune activation. Cluster_C was classified as an immune desert phenotype,characterized by immunosuppression. Further mining of molecular mechanisms in different models of glycosylation regulators. The GSVA results of Cluster_A and Cluster_B indicated that pathways like (Figure 4D), G2-M_checkpoint, PI3K/_AKT/_mTOR_signaling, inflammatory response, and Myc Target were significantly enriched in Cluster A, whereas xenobiotic metabolism, angiogenesis, and coagulation were significantly enriched in Cluster_B. The GSVA results of Cluster_ B and Cluster_C showed (Figure 4E) that G2-M checkpoint, PI3K/_ AKT/mTOR signaling, mTORC1 signaling, and other channels were highly enriched in Cluster_B, whereas interferon gamma response, coalescence, and adipogenesis were highly enriched in Cluster _ C.

### 3.4 Clinical features and transcriptomic characteristics of the glycosylation-related model

**FIGURE 5:**
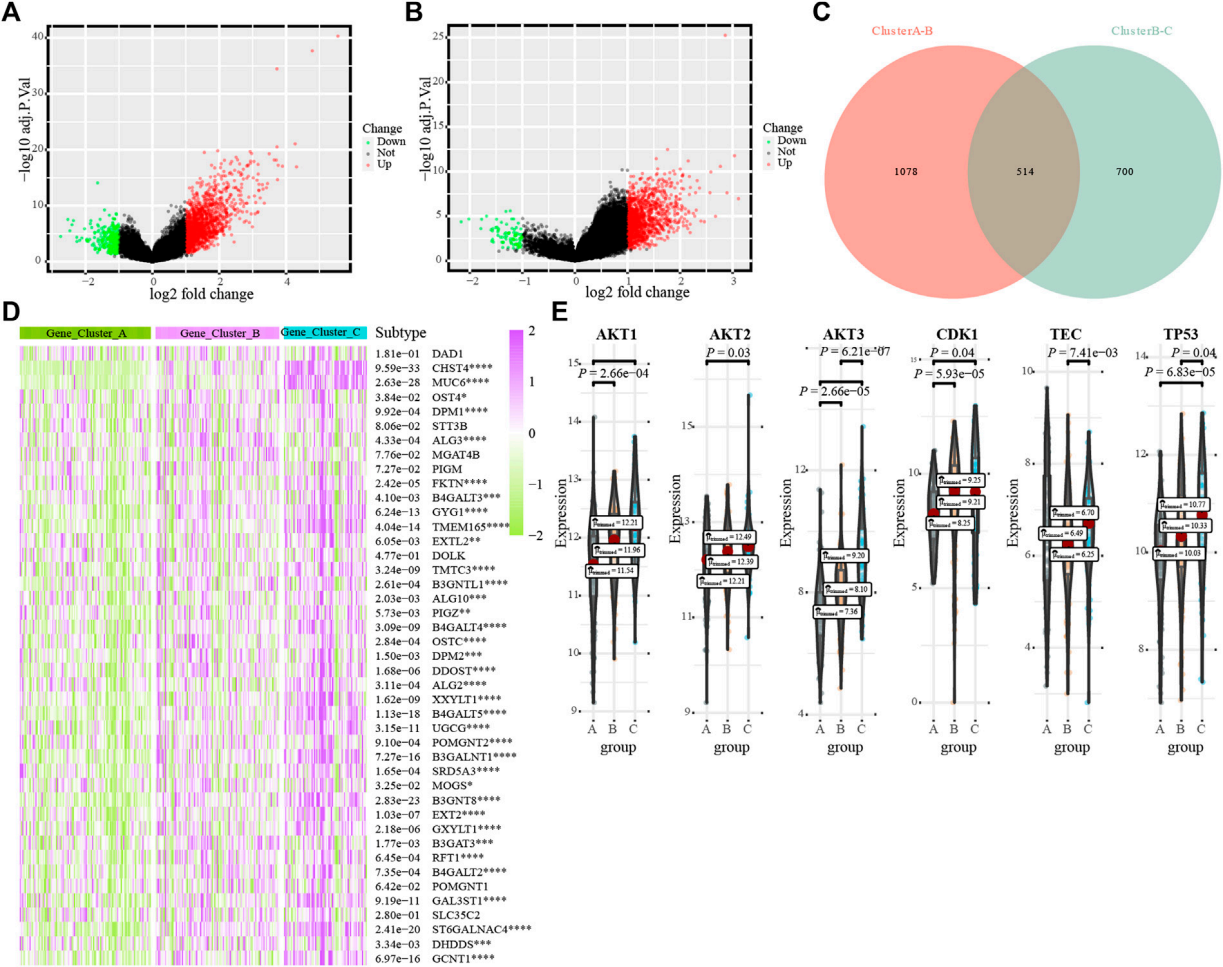
The transcriptome characterization of the glycosylation-related models. **(A)**. Distribution of differentially expressed genes between Cluster_ A and Cluster_B; **(B)**. Distribution of differentially expressed genes between Cluster_B and Cluster_C; **(C)**. Venn diagrams; **(D)**. The differential expression of 43 glycosylation regulators in different glycosylation-related subtypes; **(E)**. G2M_CHECKPOINT and PI3K_AKT_MTOR_SIGNALING in different glycosylation subtypes.

We verified the glycosylation regulatory model by constructing a glycosylation-related model. First, the limma package was used to analyze differentially expressed genes between different glycosylation regulatory gene subtypes. The screening criteria were | log(FC) | >1 and *p* < 0.05. In total, 1,592 differentially expressed genes were obtained from Cluster_A and Cluster_B (Figure 5A). In total, 1,214 differentially expressed genes were obtained from Cluster_B and Cluster_C (Figure 5B). The intersection of these two yielded 514 differentially expressed genes (Figure 5C). Univariate Cox regression analysis of the 514 differentially expressed genes revealed 79 prognosis-related glycosylation-related genes (Supplementary Table S2). PCA was applied to construct a glycosylation-related model, and the two-group model could significantly distinguish patients (Supplementary Figures S1A–C), whereas the survival of the four-group model was not significantly different (Supplementary Figure S1F), and the three-group model was more detailed than the two-group model (Supplementary Figures S1D, E). Therefore, the three-group model was the optimal typing (Gene_Cluster_A, Gene_Cluster_B and Gene_Cluster_C). Analysis of the expression of 56 prognostis-related glycosylation-regulatory genes revealed that all genes, except *DAD1*, had the highest expression in Gene_Cluster_C and the lowest expression in Gene_Cluster_A (Figure 5D). The GAVA analysis of the glycosylation regulatory model found that G2-M checkpoint and PI3K/AKT/mTOR signaling were significantly enriched in different models; therefore, we analyzed the expression of G2-M checkpoint markers (p53, CDK1) and PI3K/AKT/mTOR signaling markers (AKT1, AKT2, AKT3, and TEC) in different glycosylation-related models. The expression of key molecules of PI3K/AKT/mTOR signaling, including AKT1, AKT2, and AKT3, were highest in gene_cluster_C and lowest in gene _ cluster _A (Figure 5E). However, p53 and CDK1 showed the opposite trend. Analysis of immune cell infiltration in glycosylation-related models revealed that most immune cells were mainly enriched in Gene_Cluster_C and were low in Gene_Cluster_A (Supplementary Figure S2A). The distribution of immune checkpoints among the three glycosylation-related subtypes showed a consistent trend (Supplementary Figure S2B).

### 3.5 Clinical characteristics of the glycosylation score

**FIGURE 6:**
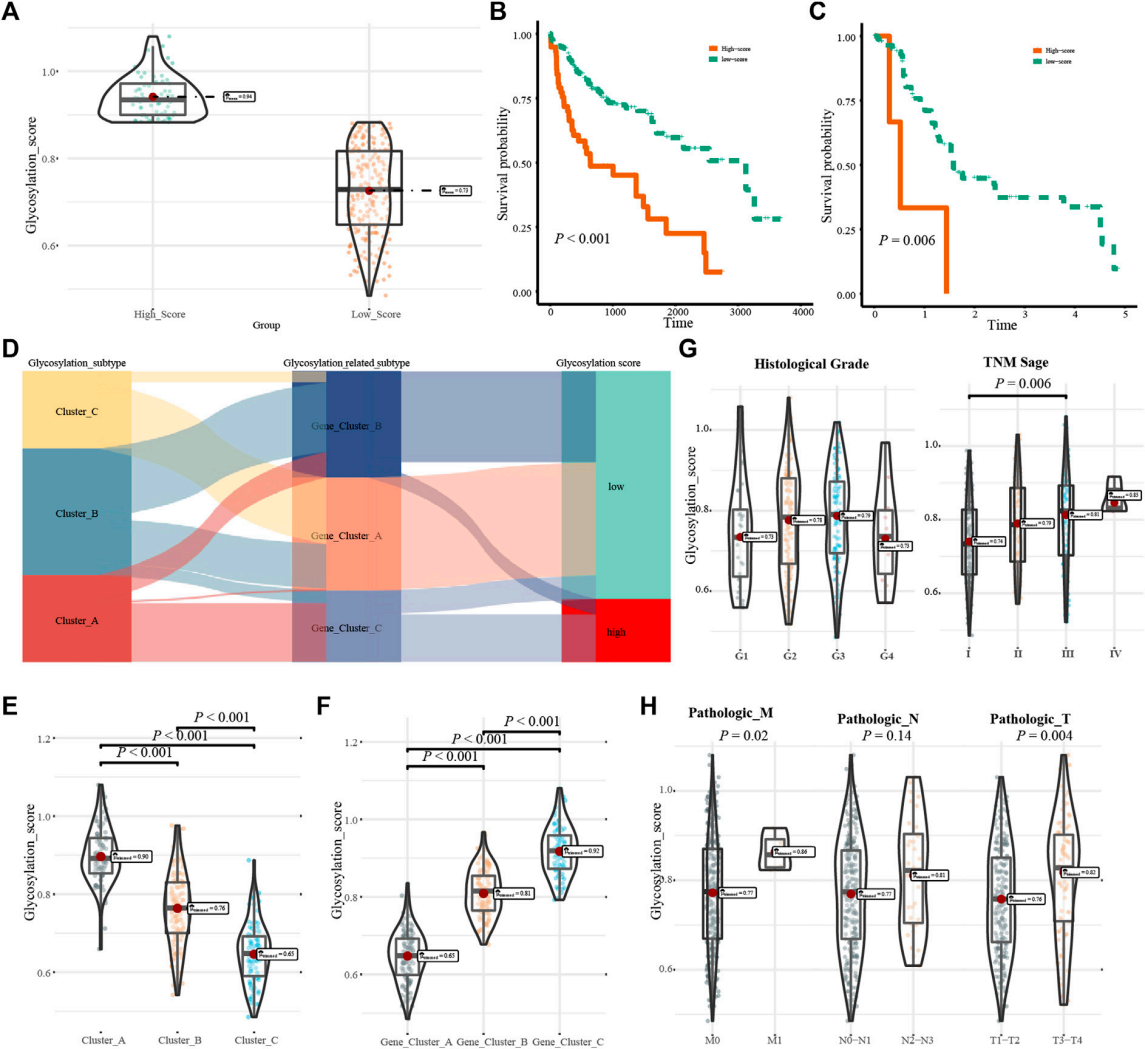
Clinical characteristics of the glycosylation score. **(A)**. Group information of glycosylation scores; **(B)**. Kaplan-Meier analysis of the high- and low-score in the TCGA-LIHC cohort; **(C)**. Kaplan-Meier analysis of high- and low-score in the GSE76427 cohort; **(D)**. Correspondence between glycosylation regulatory gene model, glycosylation-related model and glycosylation score; **(E**,**F)**. Relations between glycosylation regulator model, glycosylation-related model and glycosylation score; **(G)**. The relationship fo differentiation grade and TNM stage with glycosylation score; **(H)**. The relationship of tumor size, lymph node metastasis, and distant metastasis with glycosylation score.

Glycosylation scores were calculated for each HCC patient, and all patients were divided into high-score and low-score groups (Figure 6A). Analysis of the survival value of the glycosylation scores showed that patients with high scores had a significantly worse prognosis (*p* < 0.001, Figure 6B). When we verified the prognostic value of the glycosylation score in the GSE76427 cohort, we found that its prognostic value was high (Figure 6C). Clinical subgroup analysis was used to determine whether the glycosylation score had an independent prognostic value and found that the glycosylation score had a high prognostic value in multiple subgroups of age, sex, and TNM stage (Supplementary Figure S3). Analysis of the corresponding relationship between the glycosylation regulation model, glycosylation-related gene model, and glycosylation score revealed that most patients with high glycosylation scores corresponded to Cluster_ A, whereas the low glycosylation score included clusters_ B, Cluster_ C, and a small number of Clusters_ A members (Figure 6D). Interestingly, we found that there were significant differences in glycosylation scores between different genotypes of the glycosylation regulatory gene model and the glycosylation-related gene model (Figures 6E,F). This further explains the relationship between the glycosylation regulatory gene model, glycosylation-related gene model, and glycosylation score. By exploring the relationship between glycosylation score, differentiation grade, and TNM stage, it was found that the glycosylation score was significantly correlated with TNM stage but not with the differentiation grade of patients (Figure 6G). Further analysis of the relationship between the glycosylation score and tumor size, lymph node metastasis, and distant metastasis in TNM stage showed that the glycosylation score was mainly related to distant metastasis and tumor size (Figure 6H).

### 3.6 Screening of drug sensitivity based on the glycosylation regulatory gene model

**FIGURE 7:**
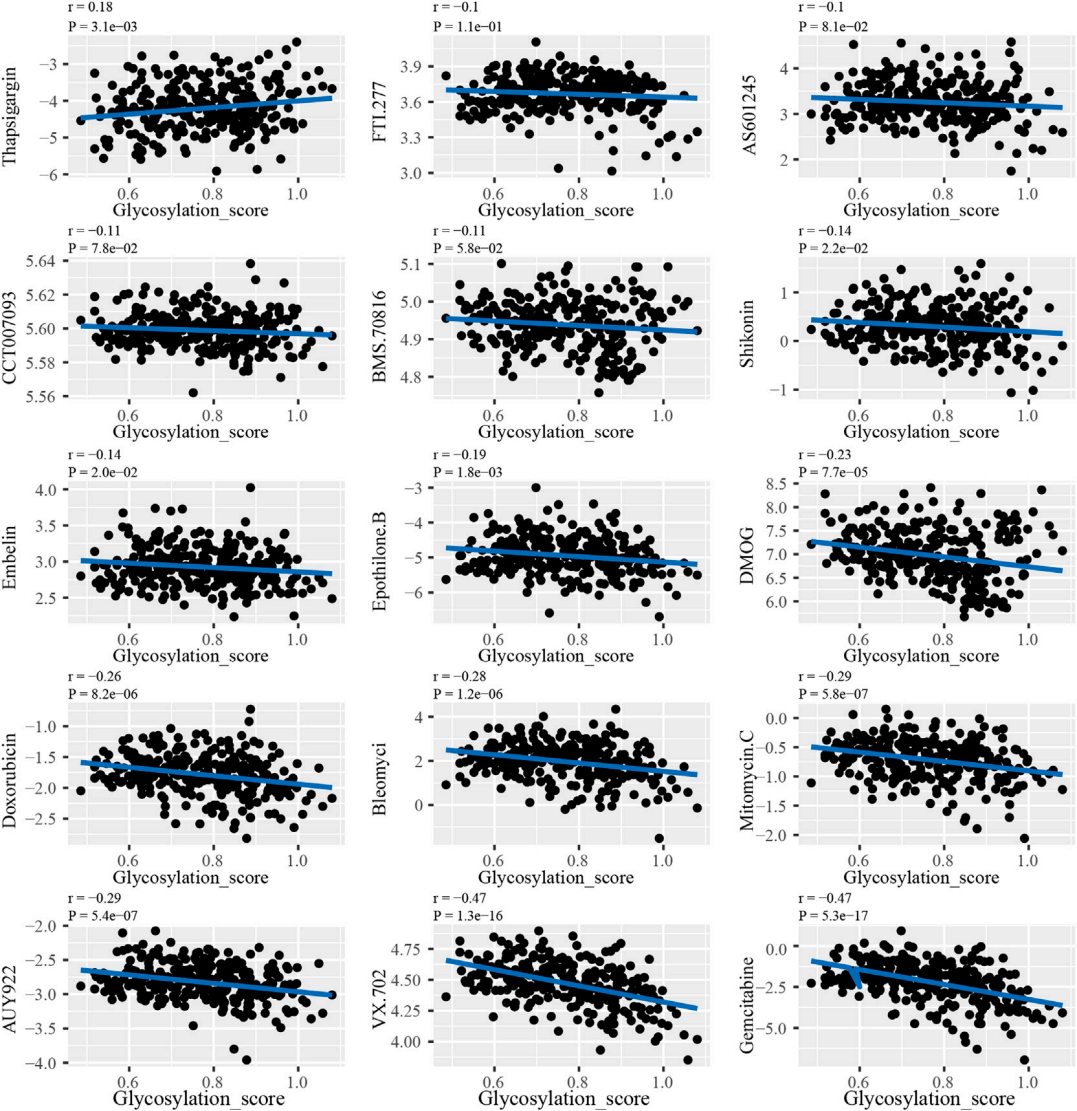
The correlation analysis between glycosylation score and drug sensitivity.

The results of analyzing the drug sensitivity of patients with different subtypes of glycosylation regulatory gene models indicate that (Supplementary Figure S3), patients in Cluster_A were most sensitive to drugs such as AS601245, AUY922, BleomyciBMS.70816, CCT007093, DMOG, Doxorubicin, Embelin, Epothilone. B, FTI.277, Gemcitabine, Mitomycin. C, Shikonin, Thapsigargin and VX.702. To ensure the accuracy of the findings, Pearson’s correlation was used to analyze the correlation between drug sensitivity and the glycosylation score (Figure 7). The glycosylation score was positively correlated with the IC_50_ of Thapsigargin (r = 0.18, *p* = 3.1e − 03), whereas it was negatively correlated with Shikonin (r = − 0.14, *p* = 2.2E − 02), embelin (r = − 0.14, *p* = 2.0e − 02), Epothilone B (r = −0.19, *p* = 1.8e−03), DMOG (r = −0.23, *p* = 7.7e−05), doxorubicin (r = −0.26, *p* = 8.2e−06), bleomyci (r = −0.28, *p* = 1.2e−06), and mitomycin. C (r = −0.29, *p* = 5.8e−07), AUY922 (r = −0.29, *p* = 5.4e−07), VX.702 (r = − 0.47, *p* = 1.3e − 16) and gemcitabine (r = − 0.47, *p* = 5.3e − 17).

## 4 Discussion

An increasing number of studies have shown that various glycosylation regulators play an indispensable role in immune microenvironment regulation, cell proliferation, and tumor drug resistance in HCC by regulating the glycosylation of proteins. At present, most studies have only focused on a single glycosylation regulator and the glycosylation regulator model of HCC has not been comprehensively described. To bridge this gap, we analyzed the glycosylation regulator model of HCC based on genomic data, hoping to help in the formulation of immunotherapy strategies.

In this study, 43 prognostic genes with differential expression in HCC were analyzed based on 255 glycosylation regulators in all tumors. Based on this, three glycosylation regulation models were revealed, where cluster_ A corresponded to the immune rejection phenotype, cluster_ B corresponded to the immunoinflammatory phenotype, and cluster_ C corresponded to the immune desert phenotype. Immune-exclusive HCC is not without the infiltration of immune cells, but these HCC cells escape the detection and destruction of immune cells *via* immunogenic shaping (Lindblad et al., 2021). In the process of immune escape in HCC, the IL-6 JAK/STAT3 signaling pathway and MYC pathway play an important role, in which IL6/JAK/STAT3 mainly promotes the glycosylation of PD-L1 to increase its stability (Zhou et al., 2020). MYC mediates the immune escape of HCC cells, mainly *via* the β-catenin protein encoded by the *CTNNB1* gene (Luke et al., 2019). In this study, we found that the prognosis of patients in cluster_B was the best; therefore, our results are consistent with these previous results, which validates the reliability of the glycosylation regulator model. The infiltration characteristics of immune cells and the prognosis of patients with immune rejection and immune desert HCC highly corresponded to cluster_ A and cluster_ C, which further proves the importance of the glycosylation regulatory gene model in HCC. We also constructed nomograms based on the glycosylation regulatory gene model. Clinicians can not only predict the prognosis of patients with HCC based on the nomogram, but can also use it to formulate clinical treatment strategies.

Differentially expressed genes are considered to be the characteristic genes of the phenotype; therefore, this study verified the glycosylation regulatory model based on the differentially expressed genes between different glycosylation regulatory gene models. Similar to the glycosylation regulatory model, we could divide HCC patients into three subtypes based on differentially expressed genes. Using enrichment analysis of immune and stromal cells, we found that these three gene subtypes were significantly related to the enrichment of immune and stromal cells. This also proves that the glycosylation regulatory gene model has different characteristics of immune cell infiltration. To determine the influence of individual differences in patients with HCC on the glycosylation regulator model, we constructed a glycosylation score. Patients in cluster_A, characterized by immune rejection, all corresponded to high glycosylation scores, whereas patients in cluster_B, characterized by immune inflammation, mostly corresponded to low glycosylation scores. In addition, we found that both G2-M checkpoint and PI3K/AKT/mTOR signaling differed significantly between different glycosylation regulatory models. Both G2-M checkpoint and PI3K/AKT/mTOR signaling differed significantly between glycosylation-regulatory gene subtypes (Sheng et al., 2021). Related studies have shown that LARP4B, OGDHL, and miR-454-3p may affect the prognosis of patients with HCC *via* G2-M checkpoint (Li et al., 2019a; Li et al., 2019b; Jiao et al., 2019). Glycosylation is closely associated with HCC cell proliferation (Takahashi et al., 2020). Therefore, we inferred that glycosylation-regulated genotypes have different cell proliferation characteristics.

We screened the drug sensitivity in HCC patients based on the glycosylation regulatory model and glycosylation score, and found drugs such as thapsigargin, shikonin, embelin, epothilone B, DMOG, doxorubicin, bleomycin, mitomycin C, AUY922, VX.702, and gemcitabine. Among them, the roles of thapsigargin, shikonin, embelin, doxorubicin, bleomycin, mitomycin C, and gemcitabine drugs in HCC have long been reported (You et al., 1994; Newell et al., 2010; Zhang et al., 2020b; Djokic et al., 2020; Diggs et al., 2021; Song et al., 2021; Cheng et al., 2022), and there are no clear studies on the indications for these drugs. In this study, we evaluated the sensitivity of these drugs on different subtypes based on the glycosylation regulatory model. We also analyzed the drugs associated with glycosylation scores to ensure the accuracy of the analysis. Thus, we provide the scope for the application of these drugs after identification of the subtypes of patients with HCC.

In conclusion, the glycosylation regulatory gene model constructed in this study can comprehensively evaluate the characteristics of immune cell infiltration in HCC patients and will facilitate guidance for clinical treatment. In addition, we constructed the glycosylation score for HCC, which had an independent prognostic value. More importantly, we screened drug sensitivity for patients with HCC based on the glycosylation regulatory gene model and glycosylation score. Our results provide a new approach for improving the clinical treatment of patients with HCC and promoting individualized HCC treatment.

## Data Availability

The original contributions presented in the study are included in the article/Supplementary Material, further inquiries can be directed to the corresponding author.

## Abbreviations

HCC, Hepatocellular carcinoma; TCGA, the Cancer Genome Atlas; CNV, copy number variation; GEO, Gene Expression Omnibus; PCA, Principal component analysis; GSVA, Gene Set Variation Analysis; GDSC, Genomics of Drug Sensitivity in Cancer.
