# Supplementary material for: Characterization of glycosylation regulator-mediated glycosylation modification patterns and tumor microenvironment infiltration in hepatocellular carcinoma
Source: Front Genet. 2022 Nov 11;13:1001901. doi: 10.3389/fgene.2022.1001901 (PMC9697576; doi:10.3389/fgene.2022.1001901)
Supplement: Supplementary file 1 [file DataSheet1.docx]

Supplementary Material

## Supplementary Figures


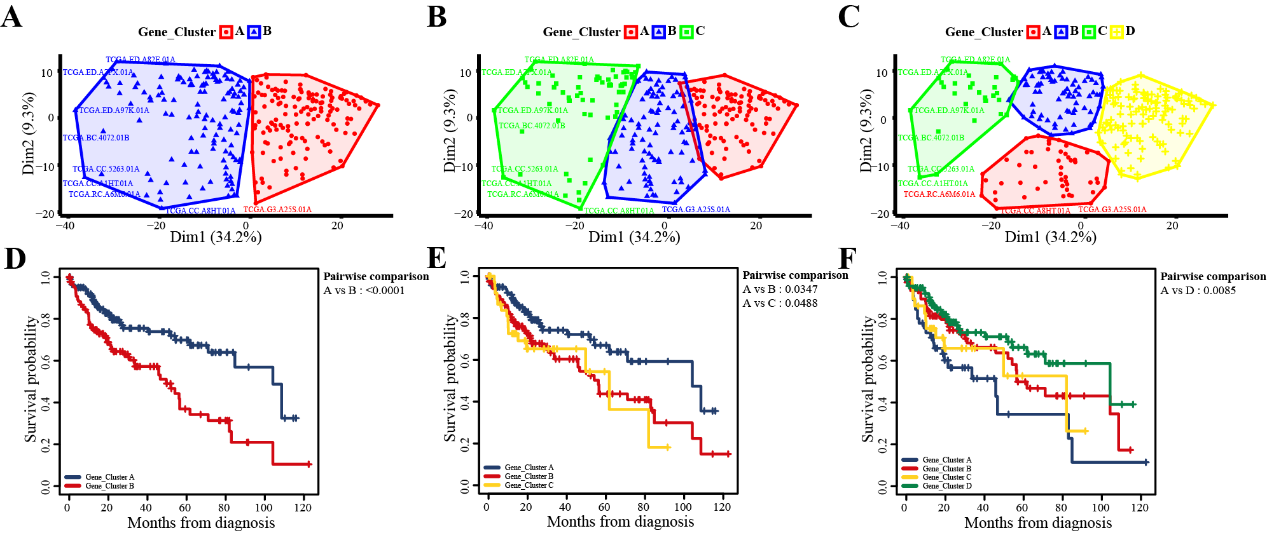


**Figure 1S.** Construction of glycosylation-related models. A-C：The PCA of two-group subtype, three-group subtype and four-group subtype; D-F: The Kaplan-Meier of two-group subtype, three-group subtype and four-group subtype.

**
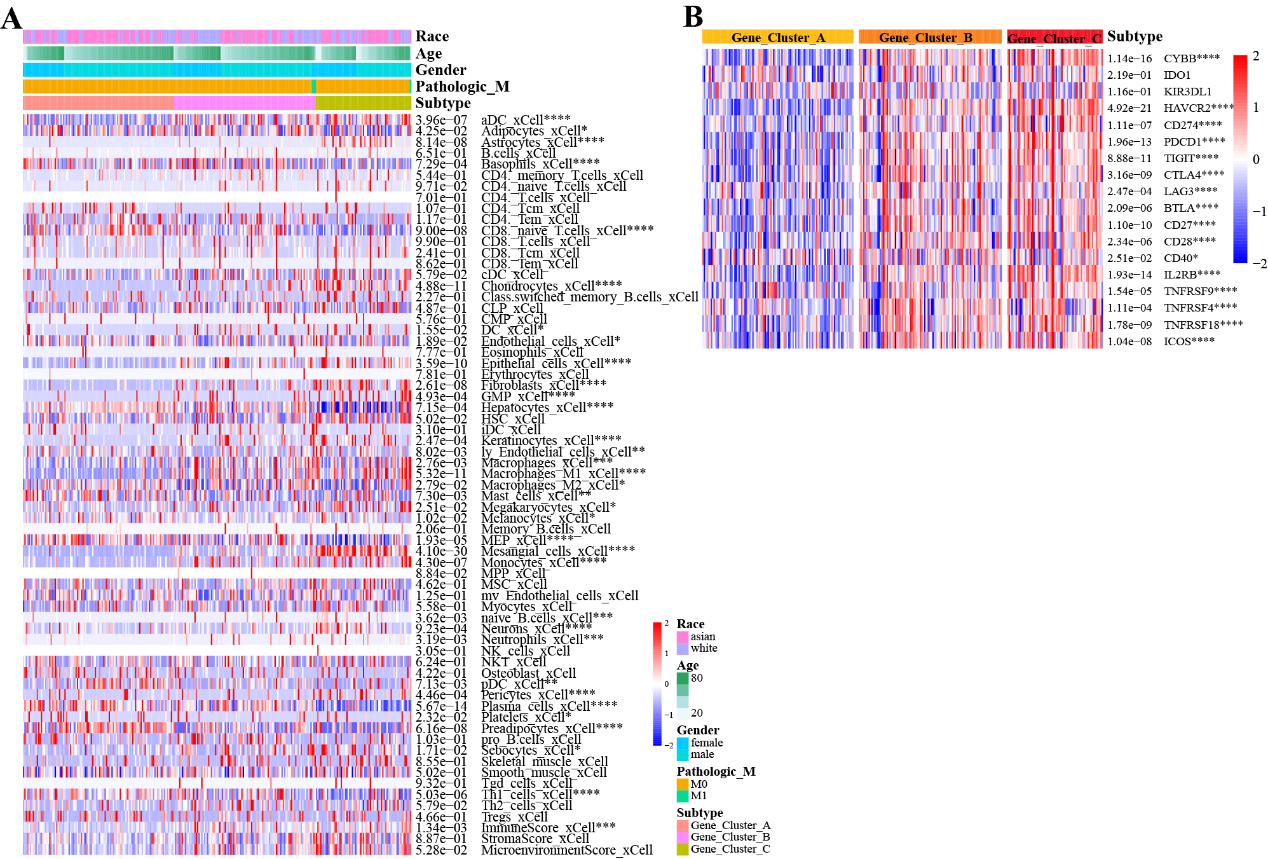
**

**Figure S2.** Immune cell and stromal cell characteristics in glycosylation-related subtypes. A. Differences in immune cell and stromal cell enrichment between glycosylation-related subtypes; B. Differences in the expression of immune checkpoints between glycosylation-related subtypes.

**
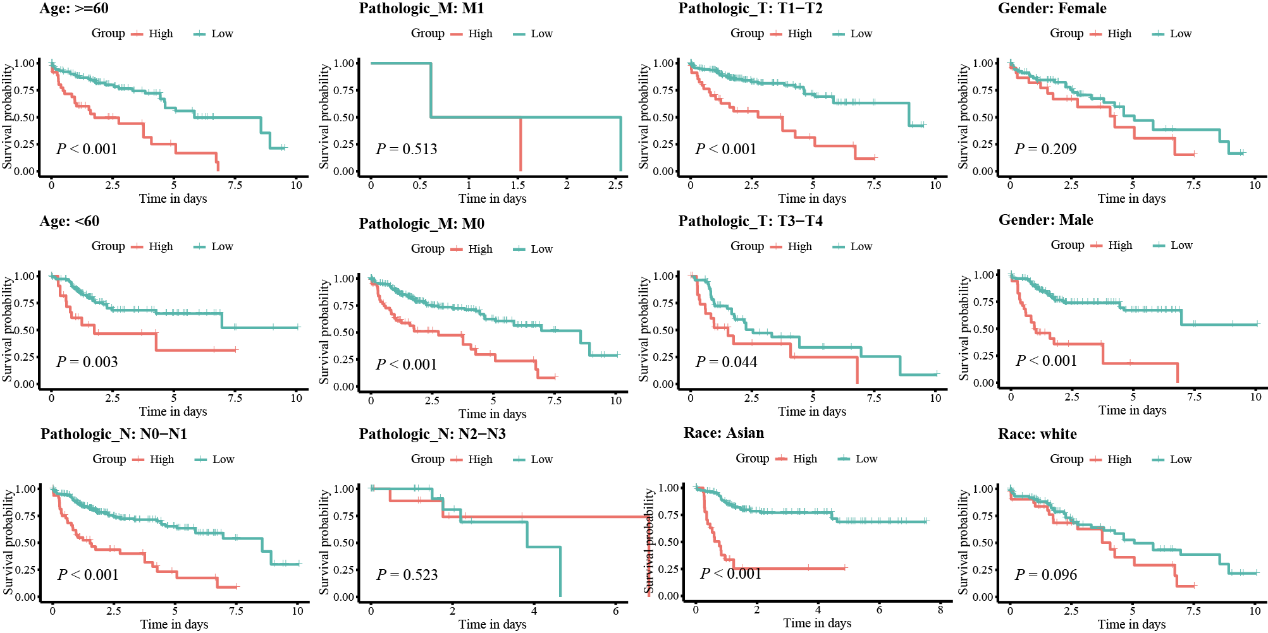
**

**Figure S3.** Kaplan-Meier analysis in the clinical subgroups.

**
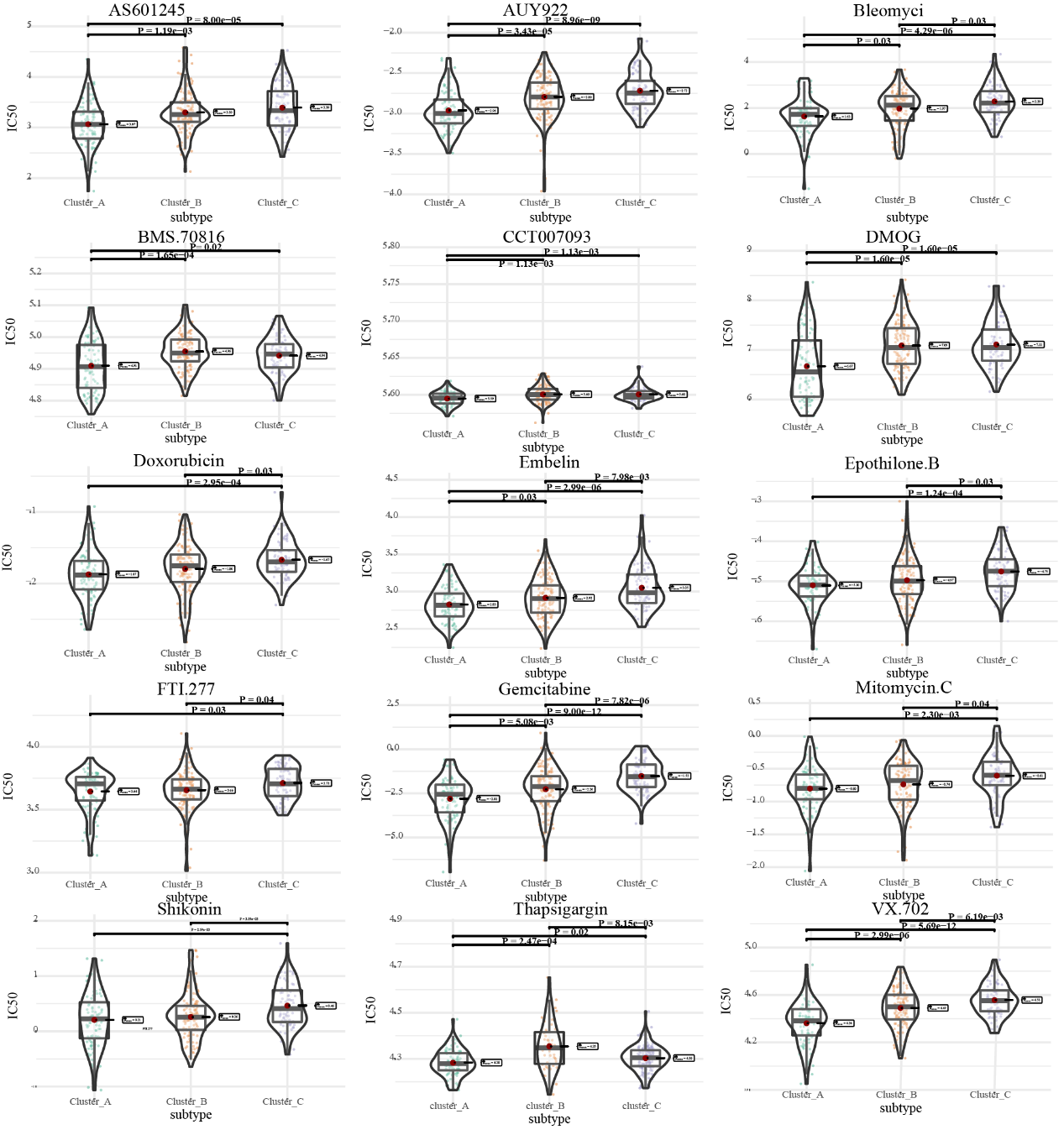
**

**Figure S4.** The differences of IC50 between glycosylation regulated gene subtypes.

**Figure Legends**

Figure 1. Flow-process diagram

Figure 2. Genetic variation in glycosylation-regulatory genes in HCC. A. Univariate Cox regression analysis of glycosylation regulatory genes; B. Differential expression of glycosylation regulated genes with prognostic value; C. Location of 43 differentially expressed genes with prognostic value on the chromosome; E. CNV of glycosylation regulators.

Figure 3. Glycosylation regulatory gene model. A. Conditional probability survival curve; B. Apply consensus clustering to construct a glycosylation regulator gene model; C. Kaplan-Meier survival analysis of the glycosylation regulator gene model; D. Calibration curve at 5 and 8 years; E. Nomogram.

Figure 4. Annotation of the glycosylation regulatory gene models. A. Enrichment of immune cells and stromal cells in different subtypes; B. Differences in immune scores and stromal scores in different subtypes. C. Differential expression analysis of immune checkpoints in different subtypes; D. The GSVA of Cluster_A-Cluster_B; E. The GSVA of Cluster_B-Cluster_C.

Figure 5. The transcriptome characterization of the glycosylation-related models. A. Distribution of differentially expressed genes between Cluster_ A and Cluster_B; B. Distribution of differentially expressed genes between Cluster_B and Cluster_C; C. Venn diagrams; D. The differential expression of 43 glycosylation regulators in different glycosylation-related subtypes; E. G2M_CHECKPOINT and PI3K_AKT_MTOR_SIGNALING in different glycosylation subtypes.

Figure 6. Clinical characteristics of the glycosylation score. A. Group information of glycosylation scores; B. Kaplan-Meier analysis of the high- and low-score in the TCGA-LIHC cohort; C. Kaplan-Meier analysis of high- and low-score in the GSE76427 cohort; D. Correspondence between glycosylation regulatory gene model, glycosylation-related model and glycosylation score; E, F. Relations between glycosylation regulator model, glycosylation-related model and glycosylation score; G. The relationship fo differentiation grade and TNM stage with glycosylation score; H. The relationship of tumor size, lymph node metastasis, and distant metastasis with glycosylation score.

Figure 7. The correlation analysis between glycosylation score and drug sensitivity.

**Supplementary Tables**

**Table S1.** The list of glycosylation-regulated genes

| genes |
| --- |
| A4GALT |
| A4GNT |
| ABO |
| ALG1 |
| ALG10 |
| ALG10B |
| ALG11 |
| ALG12 |
| ALG13 |
| ALG14 |
| ALG1L |
| ALG1L2 |
| ALG2 |
| ALG3 |
| ALG5 |
| ALG6 |
| ALG8 |
| ALG9 |
| ASGR1 |
| ASGR2 |
| B3GALNT1 |
| B3GALNT2 |
| B3GALT1 |
| B3GALT2 |
| B3GALT4 |
| B3GALT5 |
| B3GALT6 |
| B3GAT1 |
| B3GAT2 |
| B3GAT3 |
| B3GLCT |
| B3GNT2 |
| B3GNT3 |
| B3GNT4 |
| B3GNT5 |
| B3GNT6 |
| B3GNT7 |
| B3GNT8 |
| B3GNT9 |
| B3GNTL1 |
| B4GALNT1 |
| B4GALNT2 |
| B4GALNT3 |
| B4GALNT4 |
| B4GALT1 |
| B4GALT2 |
| B4GALT3 |
| B4GALT4 |
| B4GALT5 |
| B4GALT6 |
| B4GALT7 |
| B4GAT1 |
| C1GALT1 |
| C1GALT1C1 |
| C1GALT1C1L |
| CHPF |
| CHPF2 |
| CHST4 |
| CHSY1 |
| CHSY3 |
| COLGALT1 |
| COLGALT2 |
| CRPPA |
| CSGALNACT1 |
| CSGALNACT2 |
| DAD1 |
| DDOST |
| DERL3 |
| DHDDS |
| DOLK |
| DOLPP1 |
| DPAGT1 |
| DPM1 |
| DPM2 |
| DPM3 |
| ENTPD5 |
| EOGT |
| EXT1 |
| EXT2 |
| EXTL1 |
| EXTL2 |
| EXTL3 |
| FKRP |
| FKTN |
| FUT1 |
| FUT10 |
| FUT11 |
| FUT2 |
| FUT3 |
| FUT4 |
| FUT5 |
| FUT6 |
| FUT7 |
| FUT8 |
| FUT9 |
| GAL3ST1 |
| GALNT1 |
| GALNT10 |
| GALNT11 |
| GALNT12 |
| GALNT13 |
| GALNT14 |
| GALNT15 |
| GALNT16 |
| GALNT18 |
| GALNT2 |
| GALNT3 |
| GALNT4 |
| GALNT5 |
| GALNT6 |
| GALNT7 |
| GALNT8 |
| GALNT9 |
| GALNTL6 |
| GBGT1 |
| GCNT1 |
| GCNT2 |
| GCNT3 |
| GCNT4 |
| GCNT7 |
| GFPT1 |
| GFPT2 |
| GLT1D1 |
| GLT8D1 |
| GLT8D2 |
| GORASP1 |
| GTDC1 |
| GXYLT1 |
| GXYLT2 |
| GYG1 |
| GYG2 |
| GYS1 |
| GYS2 |
| HAS1 |
| HAS2 |
| HAS3 |
| IL15 |
| KRTCAP2 |
| LARGE1 |
| LARGE2 |
| LFNG |
| LMAN1 |
| MAGT1 |
| MAN1C1 |
| MCFD2 |
| MFNG |
| MGAT1 |
| MGAT2 |
| MGAT3 |
| MGAT4A |
| MGAT4B |
| MGAT4C |
| MGAT4D |
| MGAT5 |
| MGAT5B |
| MOGS |
| MPDU1 |
| MUC1 |
| MUC12 |
| MUC13 |
| MUC15 |
| MUC16 |
| MUC17 |
| MUC19 |
| MUC2 |
| MUC20 |
| MUC21 |
| MUC3A |
| MUC4 |
| MUC5AC |
| MUC5B |
| MUC6 |
| MUC7 |
| MUCL1 |
| MVD |
| NUDT14 |
| NUS1 |
| OGA |
| OGT |
| OST4 |
| OSTC |
| PGM3 |
| PIGA |
| PIGB |
| PIGM |
| PIGV |
| PIGZ |
| PLOD3 |
| PMM1 |
| PMM2 |
| POFUT1 |
| POFUT2 |
| POGLUT1 |
| POGLUT2 |
| POGLUT3 |
| POMGNT1 |
| POMGNT2 |
| POMK |
| POMT1 |
| POMT2 |
| PYGB |
| PYGL |
| PYGM |
| RFNG |
| RFT1 |
| RPN1 |
| RPN2 |
| RXYLT1 |
| SDF2L1 |
| SLC35C1 |
| SLC35C2 |
| SLC39A8 |
| SRD5A3 |
| ST3GAL1 |
| ST3GAL2 |
| ST3GAL3 |
| ST3GAL4 |
| ST3GAL5 |
| ST3GAL6 |
| ST6GAL1 |
| ST6GAL2 |
| ST6GALNAC1 |
| ST6GALNAC2 |
| ST6GALNAC3 |
| ST6GALNAC4 |
| ST6GALNAC5 |
| ST6GALNAC6 |
| ST8SIA1 |
| ST8SIA2 |
| ST8SIA3 |
| ST8SIA4 |
| ST8SIA5 |
| ST8SIA6 |
| STT3A |
| STT3B |
| TET1 |
| TET2 |
| TET3 |
| TMEM165 |
| TMEM258 |
| TMTC1 |
| TMTC2 |
| TMTC3 |
| TMTC4 |
| TRAK1 |
| TRAK2 |
| TUSC3 |
| UBE2J1 |
| UGCG |
| UGGT1 |
| UGGT2 |
| VEGFB |
| XXYLT1 |
| XYLT1 |
| XYLT2 |

**Table S2.** The results of the univariate cox regression analysis

| gene | HR | z | pvalue | lower | upper |
| --- | --- | --- | --- | --- | --- |
| MUC6 | 1.065599 | 2.212269 | 0.026948 | 1.007273 | 1.127303 |
| CHST4 | 1.070109 | 2.429356 | 0.015126 | 1.013178 | 1.130238 |
| WNT2 | 1.081327 | 2.00698 | 0.044752 | 1.001833 | 1.167128 |
| KCNJ16 | 1.08557 | 2.508803 | 0.012114 | 1.018124 | 1.157483 |
| B3GALT5 | 1.091558 | 2.169239 | 0.030065 | 1.008488 | 1.181471 |
| GLP2R | 1.114014 | 2.487251 | 0.012873 | 1.023153 | 1.212944 |
| CXCL6 | 1.058382 | 1.966339 | 0.049259 | 1.000184 | 1.119966 |
| PNMA2 | 1.143202 | 2.523395 | 0.011623 | 1.030334 | 1.268434 |
| GJB3 | 1.127054 | 3.034427 | 0.00241 | 1.043262 | 1.217577 |
| IL20RA | 1.077972 | 2.204306 | 0.027503 | 1.008357 | 1.152393 |
| SAMD12 | 1.101781 | 2.213421 | 0.026869 | 1.011161 | 1.200522 |
| TPBG | 1.101632 | 2.026755 | 0.042687 | 1.003195 | 1.209729 |
| RP11-43F13.3 | 1.119672 | 2.801721 | 0.005083 | 1.034544 | 1.211804 |
| NRG3 | 1.127896 | 2.861597 | 0.004215 | 1.038649 | 1.224811 |
| SGPP2 | 1.088737 | 2.031194 | 0.042235 | 1.002986 | 1.18182 |
| PTGS1 | 1.211523 | 2.962056 | 0.003056 | 1.067068 | 1.375534 |
| SNCA | 1.129491 | 2.105374 | 0.035259 | 1.008445 | 1.265065 |
| MMP7 | 1.072122 | 2.324674 | 0.020089 | 1.010985 | 1.136955 |
| LAMA1 | 1.083126 | 2.059263 | 0.039469 | 1.003858 | 1.168653 |
| CXCL1 | 1.092068 | 2.6272 | 0.008609 | 1.02262 | 1.166232 |
| EFNA5 | 1.109121 | 2.715146 | 0.006625 | 1.029225 | 1.19522 |
| VEPH1 | 1.086217 | 2.045489 | 0.040807 | 1.003464 | 1.175796 |
| C3orf52 | 1.15457 | 3.440414 | 0.000581 | 1.063801 | 1.253085 |
| FCHO1 | 1.113092 | 2.033088 | 0.042044 | 1.003861 | 1.234207 |
| AC005083.1 | 1.128723 | 2.446593 | 0.014421 | 1.024377 | 1.243698 |
| PFKFB3 | 1.162322 | 2.578001 | 0.009937 | 1.036719 | 1.303143 |
| MBOAT2 | 1.133312 | 2.111547 | 0.034725 | 1.009024 | 1.272908 |
| PKP3 | 1.069633 | 1.988543 | 0.046752 | 1.000968 | 1.143009 |
| SERPINE1 | 1.1244 | 2.332 | 0.019701 | 1.018881 | 1.240846 |
| TREM1 | 1.181473 | 3.300877 | 0.000964 | 1.070091 | 1.304449 |
| TNFRSF11B | 1.170463 | 3.396821 | 0.000682 | 1.068846 | 1.28174 |
| SLC6A19 | 1.095935 | 3.019064 | 0.002536 | 1.032658 | 1.163088 |
| C3orf80 | 1.106204 | 1.982134 | 0.047464 | 1.00113 | 1.222307 |
| C10orf90 | 1.094229 | 2.092186 | 0.036422 | 1.005707 | 1.190542 |
| IL1B | 1.130481 | 2.186657 | 0.028768 | 1.012796 | 1.261841 |
| CLEC5A | 1.17527 | 3.156065 | 0.001599 | 1.063117 | 1.299253 |
| GULP1 | 1.095051 | 2.487458 | 0.012866 | 1.019442 | 1.176267 |
| EPHB3 | 1.103534 | 2.045319 | 0.040823 | 1.00412 | 1.21279 |
| NPTX2 | 1.062681 | 1.997421 | 0.045779 | 1.001141 | 1.128004 |
| CXCL5 | 1.088733 | 2.782247 | 0.005398 | 1.025444 | 1.155929 |
| PRSS16 | 1.090988 | 2.732934 | 0.006277 | 1.024936 | 1.161296 |
| FHOD3 | 1.117921 | 2.77483 | 0.005523 | 1.033276 | 1.209499 |
| HTRA3 | 1.111467 | 2.230367 | 0.025723 | 1.012895 | 1.219631 |
| CA9 | 1.09504 | 3.316911 | 0.00091 | 1.037841 | 1.155391 |
| CERCAM | 1.114544 | 2.113496 | 0.034558 | 1.007909 | 1.232461 |
| CHRDL1 | 1.078756 | 2.261073 | 0.023755 | 1.010147 | 1.152025 |
| MYH11 | 0.894314 | -2.08711 | 0.036879 | 0.805258 | 0.993219 |
| MBOAT4 | 1.144423 | 2.695196 | 0.007035 | 1.037486 | 1.262383 |
| CXCL8 | 1.119674 | 2.980853 | 0.002874 | 1.039473 | 1.206064 |
| SLCO4C1 | 1.088281 | 2.746673 | 0.00602 | 1.024527 | 1.156001 |
| EVC | 1.08549 | 2.045886 | 0.040768 | 1.003451 | 1.174237 |
| FXYD2 | 1.058742 | 2.074528 | 0.03803 | 1.003157 | 1.117407 |
| LAMP5 | 1.112812 | 2.69851 | 0.006965 | 1.029687 | 1.202648 |
| ZFPM2-AS1 | 1.111912 | 2.937697 | 0.003307 | 1.035937 | 1.19346 |
| SSTR2 | 1.135061 | 2.617574 | 0.008856 | 1.032339 | 1.248004 |
| MMP16 | 1.139529 | 2.602698 | 0.009249 | 1.032781 | 1.257311 |
| CYP7A1 | 0.928062 | -2.73698 | 0.006201 | 0.879749 | 0.979028 |
| EPO | 1.125182 | 3.598735 | 0.00032 | 1.055178 | 1.199831 |
| TUSC3 | 1.105603 | 2.134028 | 0.032841 | 1.008222 | 1.21239 |
| PLEKHB1 | 1.091989 | 2.278031 | 0.022725 | 1.012363 | 1.177877 |
| ILDR1 | 1.096029 | 2.430131 | 0.015093 | 1.017899 | 1.180156 |
| TMPRSS4 | 1.133484 | 2.683743 | 0.00728 | 1.034369 | 1.242097 |
| TFF2 | 1.090475 | 2.851829 | 0.004347 | 1.027457 | 1.157358 |
| ITGB6 | 1.101402 | 2.14109 | 0.032267 | 1.008204 | 1.203215 |
| POF1B | 1.108101 | 3.087573 | 0.002018 | 1.0382 | 1.18271 |
| CREB3L1 | 1.095465 | 2.035549 | 0.041796 | 1.003391 | 1.195987 |
| CYP26B1 | 1.206247 | 4.302321 | 1.69E-05 | 1.107483 | 1.313818 |
| TRIM54 | 1.123589 | 3.077315 | 0.002089 | 1.043218 | 1.210151 |
| LHFPL3-AS2 | 1.082563 | 2.079485 | 0.037573 | 1.00457 | 1.166611 |
| CTAG2 | 1.059729 | 2.297194 | 0.021608 | 1.008553 | 1.113501 |
| CHGA | 1.113849 | 2.914257 | 0.003565 | 1.035938 | 1.197621 |
| FRAS1 | 1.092602 | 2.510563 | 0.012054 | 1.019613 | 1.170817 |
| C6 | 0.909745 | -2.17332 | 0.029756 | 0.835357 | 0.990757 |
| CLEC2L | 1.142091 | 4.021043 | 5.79E-05 | 1.070473 | 1.218499 |
| MEP1A | 1.089661 | 3.025049 | 0.002486 | 1.030694 | 1.152002 |
| AKR1B15 | 1.074445 | 2.421343 | 0.015463 | 1.013776 | 1.138745 |
| TINAG | 1.056508 | 2.10295 | 0.03547 | 1.003745 | 1.112046 |
| LINC01370 | 1.081386 | 2.7904 | 0.005264 | 1.023559 | 1.14248 |
| MAGEA6 | 1.065213 | 2.678637 | 0.007392 | 1.017094 | 1.115608 |
